# Sex differences in murine bone matrix maturation modulate osteoblast-endothelial interactions

**DOI:** 10.1186/s13293-026-00960-3

**Published:** 2026-08-02

**Authors:** Aikta Sharma, Roger J. H. Emery, Andrew A. Pitsillides, Claire E. Clarkin

**Affiliations:** 1 Department of Mechanical Engineering, University College London, London, WC1E 7JE UK; 2 School of Biological Sciences, University of Southampton, Southampton, SO17 1BJ UK; 3 The Hamlyn Centre for Robotic Surgery, Imperial College London, London, SW7 2AZ UK; 4 Department of Comparative Biomedical Sciences, Royal Veterinary College, London, NW1 0TU UK

**Keywords:** Sex dimorphism, Raman spectroscopy, Extracellular matrix, Collagen, VEGF, Osteoblast, Endothelial cell

## Abstract

**Background:**

Bone formation during skeletal growth and repair is divergently modulated by osteoblast-derived vascular endothelial growth factor (VEGF), which contributes to the sexual dimorphism of the bone vasculature. While the extracellular matrix (ECM) provides structural and instructive cues to developing vasculature, whether the osteoblast-derived matrix contributes to this dimorphism remains unclear.

**Methods:**

Primary osteoblasts from the long bones of neonatal female and male C57BL/6J mice were cultured under basal or osteogenic conditions for compositional ECM analysis by Raman spectroscopy. Primary murine bone marrow-derived endothelial cells (BMECs) were seeded onto established osteoblast layers and maintained in heterotypic cocultures to assess contact-mediated effects of osteoblast ECM on BMEC survival and expansion. Osteoblast-derived conditioned media (CM) were used to evaluate soluble-factor contributions, with VEGF-A concentration quantified by ELISA.

**Results:**

Raman spectroscopy of monocultured osteoblasts revealed sexually dimorphic ECM signatures independent of cellular growth profiles. Female matrices were enriched with type I collagen-associated proline and hydroxyproline and octacalcium phosphate, consistent with a matrix-dominant signature. Male matrices exhibited lower levels of collagen-associated components and instead adopted a more mineral-mature profile, reflected by CAP accumulation and an elevated mineral/matrix ratio. In heterotypic cocultures, BMEC numbers were 1.39-fold higher with male than female osteoblasts. CM treatment of BMECs did not recapitulate these effects despite higher VEGF-A release from male osteoblasts.

**Conclusions:**

Sex differences in osteoblast-derived ECM are linked to divergent, contact-dependent modulation of BMEC behaviour. These findings indicate that intrinsic sex differences in osteoblast matrix maturation may contribute to sex-specific regulation of the skeletal vascular niche. Defining how osteoblast-derived ECM regulates skeletal vascularisation may reveal targets for selectively modulating pathological skeletal angiogenesis in women and men.

**Supplementary Information:**

The online version contains supplementary material available at https://doi.org/10.1186/s13293-026-00960-3.

## Abstract (plain language summary)

Bone is a sexually dimorphic organ, with women and men differing in bone size, strength and risk of fracture. The skeletal vasculature is essential for bone growth and repair, with bone forming osteoblast cells influencing blood vessel development through the skeletal extracellular matrix (ECM). Although the interactions between osteoblast and vascular cells are crucial for lifelong skeletal health, whether sex differences in bone structure between women and men arise from differences in osteoblast activity or sex differences in blood vessel growth remains unknown. Here, we show that female and male mouse osteoblasts deposit compositionally distinct ECMs that differentially influence vascular endothelial cell behaviour. Female osteoblasts produce a collagen-rich matrix with low mineral content. In contrast, male osteoblasts produce matrices containing less collagen and more mineral, while releasing elevated levels of the blood vessel-promoting factor, VEGF-A, compared with female osteoblasts. When placed directly onto these osteoblast layers, vascular cell growth was greater in cocultures with male than female osteoblasts and could not be reproduced by exposure to osteoblast-derived soluble factors alone. These findings identify a contact-dependent relationship through which sex differences in osteoblast ECM composition influence vascular cell behaviour in bone. Understanding how osteoblast-vascular interactions differ by sex may explain the variability in bone health, healing capacity and disease risk between women and men. Further, our approach may inform the discovery of new therapeutic targets that support bone growth and repair while targeting abnormal blood vessel growth in a sex-specific manner.

**Supplementary Information:**

The online version contains supplementary material available at https://doi.org/10.1186/s13293-026-00960-3.

## Highlights

Primary osteoblasts from female and male C57BL/6J mouse long bones synthesise compositionally distinct ECMs.

ECM produced by female osteoblasts contains raised type I collagen-associated components and OCP, whereas the male osteoblast ECM comprises relatively lower levels of type I collagen-associated species and CAP accumulation.

BMEC growth is markedly enhanced in heterotypic direct-contact cocultures with male than female osteoblasts.

Male osteoblasts release higher levels of the pro-angiogenic factor, VEGF-A, than female osteoblasts.

The sex-specific effects of the osteoblast ECM on BMECs are contact-dependent and are not reproduced by treatment with osteoblast-derived CM.

**Supplementary Information:**

The online version contains supplementary material available at https://doi.org/10.1186/s13293-026-00960-3.

## Introduction

The skeletal system is profoundly sexually dimorphic, with differences in bone structure, strength and geometry emerging during puberty and progressively diverging with age under the influence of gonadal sex hormones [1–4]. During skeletal growth, male bone undergoes greater periosteal apposition, resulting in greater bone diameter and cortical thickness. In contrast, during puberty, female bone experiences relative inhibition of periosteal apposition and instead increase cortical thickness through endosteal bone formation thereby reducing the endosteal perimeter. These sex-specific growth trajectories contribute to larger male bone size, increased bone strength and lower fracture rates [1–3, 5]. However, variations in body and bone size alone do not fully account for sexually dimorphic skeletal phenotypes [6] and instead suggest that additional regulatory mechanisms contribute to these differences.

Circulating sex steroids are central regulators of bone homeostasis [7, 8], yet their cellular effects are not uniform between the sexes due to the involvement of divergent receptor-mediated pathways within skeletal cell populations. Oestrogen receptor α (ERα) is a principal mediator of oestradiol-stimulated bone formation [9–13] in both sexes [14] and is involved in the regulation of strain-induced mechanotransduction signalling pathways in osteoblasts and osteocytes in vitro [15–17], whereas androgen receptor signalling is critical for skeletal accrual and maintenance in males. Increasing evidence indicates that sexual dimorphism in bone is not, however, solely hormone driven [18, 19]. Indeed, intrinsic sex differences have been reported across a number of skeletal cell types including osteoblasts [20], osteoclasts [21, 22] and osteocytes [23, 24], implying that cell-autonomous mechanisms that may be influenced by sex chromosomes, epigenetic programming or early developmental imprinting contribute to the lifelong skeletal divergence. For instance, osteoclast precursor populations exhibit sex differences, with female cells undergoing accelerated osteoclastic differentiation alongside transcriptional enrichment of genes involved in innate immune and inflammatory response pathways [21]. Osteoblasts derived from human craniosynostosis patients similarly display sex-specific differences in differentiation, with male osteoblasts showing higher early differentiation activity and reduced proliferation compared with females [25].

Bone vascularisation is essential for mineralisation, nutrient delivery and osteogenic coupling [26] and is, at least partly, regulated by osteoblast-derived vascular endothelial growth factor (VEGF) [27, 28]. We previously showed that osteoblast-specific deletion of *Vegfa* (VEGFKO), somewhat surprisingly, produces sexually dimorphic vascular and matrix phenotypes in vivo [29, 30], and that *Vegf*-deficient osteoblasts synthesise sex-specific extracellular matrices (ECMs) in vitro with impaired mineralisation in males being associated with increased vascularisation in vivo [29]. Despite advances enabling the detailed visualisation of skeletal vascular networks [31–34], whether sex differences in bone matrix composition contribute to the regulation of skeletal endothelial cell function remains poorly defined. Given the tight coupling between osteogenesis and angiogenesis [33, 35–37], we hypothesised that intrinsic sex differences in osteoblast matrix assembly influence skeletal endothelial cell behaviour. To test this, we established separate murine heterotypic primary coculture models comprising female and male osteoblasts with bone marrow-derived endothelial cells (BMECs) to determine whether sex-specific matrix composition influences vascular endothelial cell behaviour. By integrating functional coculture assays with Raman spectroscopic profiling of matrix composition, we assess whether intrinsic sexual dimorphism in osteoblast activity exists and whether these differences functionally influence endothelial cell behaviour in a sex-specific manner within a defined in vitro coculture model, thereby providing insight into mechanisms that may contribute to sexually dimorphic regulation of the skeletal vascular niche.

## Methods

### Isolation and culture of female and male osteoblasts

All mice utilised in these studies were kept in controlled conditions at the Biomedical Research Facility, University Hospital Southampton, UK (*ad libitum* access to food and water, controlled temperature (20 °C to 24 °C), humidity (55% ± 10%) and light cycles (12 h on and 12 h off)). All cell isolation procedures were performed in accordance with the UK Animals (Scientific Procedures) Act of 1986 and regulations set by the UK Home Office and local institutional guidelines. Primary long bone osteoblasts were separately isolated from 4-5-day old female and male C57BL/6J mouse littermates, obtained from three individual litters, using the collagenase-collagenase-ethylenediaminetetraacetic acid (EDTA)-collagenase sequential digest method as previously described [38]. Sex was determined by measurement of anogenital distance, which is greater in males than females [39], and by visual assessment of scrotal pigment [40]. Long bones from female and male mice were isolated and processed separately to prevent cross-contamination. Bones from mice of the same sex within each litter were pooled for each osteoblast isolation, with three independent isolations used as biological replicates.

Briefly, tibiae, fibulae and femora were isolated and washed in Hank’s balanced salt solution (HBSS; Merck, UK) prior to incubation in 1 mg/ml collagenase type II (Worthington Biochemical Corporation, USA) in HBSS for 10 min at 37 °C. The supernatant from this initial digest was discarded. The second, third and fourth fractions were retained and pooled following sequential digestion in 1 mg/ml collagenase type II for 30 min, 4 mM EDTA (Merck, UK) for 10 min and 1 mg/ml collagenase type II for 30 min, respectively, all performed at 37 °C. Isolated cells were resuspended in basal Phenol red-free α-Minimum essential medium (αMEM; Gibco, UK) containing 10% (v/v) heat-inactivated fetal bovine serum (FBS, lot 2096994; Gibco, UK ), 50 µg/ml gentamicin (Merck, UK) and 100 U/ml penicillin, 100 µg/ml streptomycin (Merck, UK), with female and male cell cultures expanded separately in 75 cm^2^ flasks. Cells were cultured until 80% confluent in a humidified incubator set at 37 °C/5% CO_2_ and used at passage 1.

At confluence, female and male osteoblasts were seeded on uncoated quartz coverslips (#No5, thickness: 0.5 mm, Ø: 20 mm; UQG-Optics, UK) at a density of 2.56 × 10^3^ cells/cm^2^, equivalent to 10,000 cells per well, and maintained in monolayers as previously described [41] in preparation for Raman spectroscopy. Quartz coverslips were used to minimise background fluorescence and enable the acquisition of Raman signal with sufficient signal-to-noise (> 3). In preparation for heterotypic direct-contact coculture, female and male osteoblasts were plated at the same density, with corresponding monoculture controls seeded at the same time. All osteoblasts were seeded in basal αMEM and left to adhere for 48 h. Following this, cultures were maintained in either basal or osteogenic medium consisting of basal αMEM supplemented with 50 µg/ml L-ascorbic acid and 2 mM β-glycerophosphate (Merck, UK) [38, 41]. Culture media were replenished every third day until day 7 of culture unless otherwise stated.

### Protein assay and VEGF-A ELISA

On day 6 of osteoblast culture, the cell culture media were aspirated and replaced with low-serum basal αMEM containing 1% (v/v) FBS. Cells were cultured for a further 24 h ahead of CM collection on day 7 of culture. The Pierce™ bicinchoninic acid protein assay kit and reagents (Thermo Fisher Scientific, USA) were used to quantify total protein concentration in cell lysates, isolated as previously described [42].

A VEGF-A mouse sandwich enzyme-linked immunosorbent assay (ELISA) kit and reagents (R&D Systems, USA) were used to quantify natural and recombinant VEGF-A isoforms (VEGF_120_ and VEGF_164_) in the collected CM as per the manufacturer’s instructions. VEGF-A concentrations quantified in the CM were normalised to total protein concentration.

### Raman spectroscopy, spectral processing and analysis

Following CM collection on day 7, female and male osteoblast cell layers were briefly washed with 1X phosphate buffered saline (PBS; Merck, UK) prior to fixation in 4% formaldehyde in preparation for Raman spectroscopy. Raman spectra were collected using an InVia^®^ Raman microscope (Renishaw, UK) equipped with a 532 nm laser (with a Gaussian beam profile) and 2400 lines/mm diffraction grating, providing a spectral resolution of ~ 1.06 cm^− 1^. Spectra were acquired using a 63× water immersion objective (Leica, Germany) with a numerical aperture of 1.2, providing a diffraction-limited spot size of 280 nm, as previously described [41]. Wavenumber and intensity calibration were performed using the 520.7 cm^− 1^ peak of an internal silicon wafer ahead of spectral acquisition. Raman spectra were acquired using single point static scans with an exposure time of 20 s, three accumulations and 100% laser power within the 800 cm^− 1^ to 1700 cm^− 1^ range of the “fingerprint region” [43] in conjunction with the WiRE 4.1 software (Renishaw, UK) and a charged-coupled device. Power at the sample was approximately 30 mW.

Raman spectra were collected from five points within the cytoplasmic region of 25 female and male osteoblasts per group across three biological replicates, corresponding to individual osteoblast isolations. To minimise spectral contamination from neighbouring cells, only osteoblasts surrounded by a clearly defined, cell-free margin were selected as the acquired Raman signal reflects the full focal volume. Cytoplasmic regions were selected while avoiding nuclei, cell borders and visible debris. Raman spectra of blank quartz (Figure S1A) were also acquired using the same spectral acquisition settings for post-acquisition background subtraction procedures.

Spectra with poor signal-to-noise (< 3) were visually identified and reacquired where necessary. Cosmic ray artefacts were removed from individual cell spectra upon acquisition, and quartz contributions were removed by manual background subtraction procedures using spectra acquired from blank quartz coverslips using the same acquisition settings as previously described [41] (Figure S1B-C). Following quartz subtraction, cell spectra were visually inspected to confirm that neither under- nor over-subtraction had occurred. Spectra acquired within each biological replicate were used to generate replicate-level class mean spectra for each sex and culture condition. Subsequent spectral preprocessing, including Haar-wavelet denoising with 6-point smoothing (Figure S1D), was performed in iRootLab [44]. Polynomial baseline correction was applied to denoised class mean spectra using 9th -order polynomial fitting (Figure S1E), with no peak model included at this stage. Spectra were then wavenumber and intensity normalised to the 1004 cm^− 1^ peak of phenylalanine (Figure S1F, S2A-D).

Second derivative analysis was performed in WiRE 4.1 on normalised class mean spectra to guide peak identification prior to spectral deconvolution, with band positions identified as negative features at their corresponding Raman shifts [45–47] (Figure S2E-H). Raman peaks and bands of interest, including those corresponding to the type I collagen-associated components (proline, 853 cm^− 1^ and hydroxyproline, 876 cm^− 1^) and mineral-associated phosphate species (amorphous calcium phosphate, ACP, 948 cm^− 1^; octacalcium phosphate, OCP, 970 cm^− 1^ and carbonated apatite, CAP, 959 cm^− 1^), were assigned based on established Raman spectroscopic literature [48–52] and identified in second derivative class mean spectra. Univariate spectral deconvolution was subsequently performed in WiRE 4.1 on the corresponding, independently processed, non-derivative biological replicate-level class mean spectra (Figure S2A-D), as previously described [41]. Mixed Lorentzian and Gaussian curves were fitted to each spectrum (Figure S2I) to extract peak areas in preparation for peak area ratio analysis and statistical testing. No peak intensities or areas were extracted from the second derivative spectra. Fitting reproducibility was determined by repeating spectral deconvolution five times for each biological replicate-level class mean spectrum, with peak parameters recorded only when reproducible fits returned χ^2^ values below 1. For multivariate analysis, spectra were pre-processed and normalised as described above and then mean-centred prior to principal component analysis (PCA), with a maximum of 10 principal components, in iRootLab [44].

### Culture of bone marrow-derived endothelial cells

Primary BMECs derived from C57BL/6J mice were purchased from Cell Biologics Inc. (Chicago, USA; C57-6221). The donor sex was not specified by the supplier. BMECs were cultured in complete endothelial cell medium (M1168) and utilised at passage 3–5, following the manufacturer’s protocols and recommendations.

### Fluorescent labelling of bone marrow endothelial cells

CellTracker™ Green 5-chloromethylfluorescein diacetate dye, prepared in dimethylsulfoxide (DMSO; Invitrogen, UK), was utilised for fluorescent labelling of BMECs following the manufacturer’s instructions. 1 × 10^6^ BMECs were labelled in suspension with CellTracker™ Green at a concentration of 5 µM in serum-free endothelial cell medium for 30 min at 37 °C. Unlabelled control cells were processed in parallel using serum-free basal endothelial cell medium containing equivalent volumes of DMSO. Unlabelled control and Cell Tracker™ Green-labelled BMECs were then diluted in complete basal endothelial cell medium then subcultured.

### Direct-contact heterotypic coculture

After 7 days of osteoblast culture, the culture media were aspirated and replaced with fresh basal αMEM containing 10% FBS (v/v) at 50% (v/v) of the original culture volume. Unlabelled and CellTracker™ Green-labelled BMECs were resuspended in an equivalent volume of complete endothelial cell medium and plated directly into wells containing female or male osteoblasts at a density of 2.56 × 10^3^ cells/cm^2^, thereby generating cocultures maintained in 50:50 osteoblast: BMEC medium (experimental timeline is presented in Figure S3A). These mixed cultures of primary osteoblasts and BMECs are hereafter referred to as heterotypic direct-contact cocultures. Heterotypic cell layers were maintained in direct-contact cocultures for up to 72 h in a humidified incubator set at 37 °C/5% CO_2_, with no further media changes after coculture establishment. The 72 h timepoint was selected as the endpoint for this series of experiments due to the substantial decay of CellTracker™ fluorescence (Figure S3B).

Coculture control wells consisted of osteoblast monocultures alongside unlabelled and CellTracker™ Green-labelled BMECs seeded at density of 2.56 × 10^3^ cells/cm^2^. BMEC monocultures were seeded in complete endothelial cell medium and left to adhere for 2 h. Following this, equivalent volumes of basal αMEM were added to generate 50:50 (v/v) osteoblast: BMEC medium, as described above. Experimental nomenclature used hereafter is as follows: pre-basal refers to osteoblasts cultured in basal medium for 7 days prior to BMEC addition, whereas pre-osteo refers to osteoblasts cultured in osteogenic medium for 7 days prior to BMEC addition (Figure S3A).

### Quantification of BMEC survival in coculture

After 24 h and 72 h of coculture, the culture media were aspirated and cell layers were briefly washed with 1X PBS prior to fixation with 4% formaldehyde. Cell nuclei were fluorescently labelled with Hoechst 33258 (Invitrogen, UK) at a concentration of 1 µg/ml for 15 min in the dark at room temperature. Cocultured cells were imaged using the Deltavision Elite fluorescence microscope (GE Healthcare Sciences, USA) in combination with the SoftWoRx software (version 6). Dual-channel images of Hoechst fluorescence (Figure S4A) and the CellTracker™ Green fluorescence (Figure S4B) were captured sequentially using the in-built DAPI bandpass filter set (excitation: 390 ± 18 nm and emission: 435 ± 48 nm) and the fluorescein isothiocyanate (FITC) bandpass filter set (excitation: 475 ± 28 nm and emission: 525 ± 48 nm), respectively. Images were acquired using a 10× air objective with numerical aperture of 0.4, providing a field of view of 1.33 mm x 1.33 mm.

Following background subtraction, intensity normalisation and pseudocolouring of paired DAPI/FITC images (Figure S4C), CellTracker™ Green-labelled BMECs were manually counted using the “multi-point” function in FIJI [53] (Figure S4D). Only CellTracker™ Green-positive cells containing a clearly visible Hoechst-positive nucleus were included in the analysis. The total number of cell nuclei in each image was determined using an automated custom in-house macro in FIJI as previously described [29]. The number of CellTracker™ Green-labelled BMECs was subtracted from the total number of nuclei in paired DAPI/FITC images from the same field of view to determine osteoblast number. The number of fluorescent BMECs was then divided by the number of osteoblasts to determine BMECs per osteoblast, prior to normalisation to the imaged field of view. A total of 40 images were acquired randomly from four individual cocultures per condition for each biological replicate.

### Growth curves

To assess osteoblast growth profiles across culture conditions over time, cell layers were fixed in 4% formaldehyde on days 1, 3, 7, 8 and 10 of culture. To monitor BMEC growth, BMEC monocultures were fixed after 24 h and 72 h of culture, corresponding to the coculture timepoints. Following fixation, all cell layers were washed with 1X PBS and cell nuclei were stained with Hoechst 33258. For each biological replicate, 40 images were acquired randomly from four individual monocultures per condition and sex for each timepoint, with cell number determined as previously described [29].

### Conditioned media collection and treatment

CM were collected from each osteoblast monoculture at the corresponding 24 h and 72 h coculture time points and used to treat unlabelled BMECs seeded at 2.56 × 10^3^ cells/cm^2^ in basal endothelial cell medium for 24 h. CM-treated BMECs were subsequently fixed with 4% formaldehyde, stained with Hoechst 33258 and imaged by fluorescence microscopy prior to growth curve analysis, as described above.

### Statistical analysis

Statistical analyses were performed in GraphPad Prism (version 9; GraphPad Software, USA) using biological replicate-level values. Unless otherwise stated, quantitative data are presented as mean ± standard deviation (SD). For Raman spectroscopic and imaging-based datasets, technical measurements acquired within each independent biological replicate were first summarised to generate a single replicate-level values for each sex, culture condition and timepoint, as appropriate, prior to statistical analysis. Repeated-measures analyses were applied where basal and osteogenic conditions, or sequential timepoints, were paired within the same independent osteoblast isolation, as cells derived from each litter were divided across experimental conditions.

Repeated-measures two-way ANOVA was used to determine the independent and interactive effects of time and culture condition on osteoblast growth profiles within each sex. Repeated-measures two-way ANOVA was also used to assess the independent and interactive effects of sex and culture condition on VEGF-A release, Raman-derived peak areas and peak area ratios, BMEC number in direct-contact cocultures with osteoblasts, and BMEC number following CM treatment. Šídák’s post hoc test was conducted for multiple comparisons following ANOVA, where appropriate. Changes in BMEC number in monocultures between 24 h and 72 h were assessed using Student’s paired t-test. The alpha level was set at 0.05, with *p* < 0.05 considered statistically significant. Significance is denoted as *p < 0.05, **p < 0.01, ***p < 0.001 and ****p < 0.0001 unless otherwise stated.

## Results

### Sex differences in osteoblast-derived VEGF-A release are independent of cellular growth profiles

**Fig. 1:**
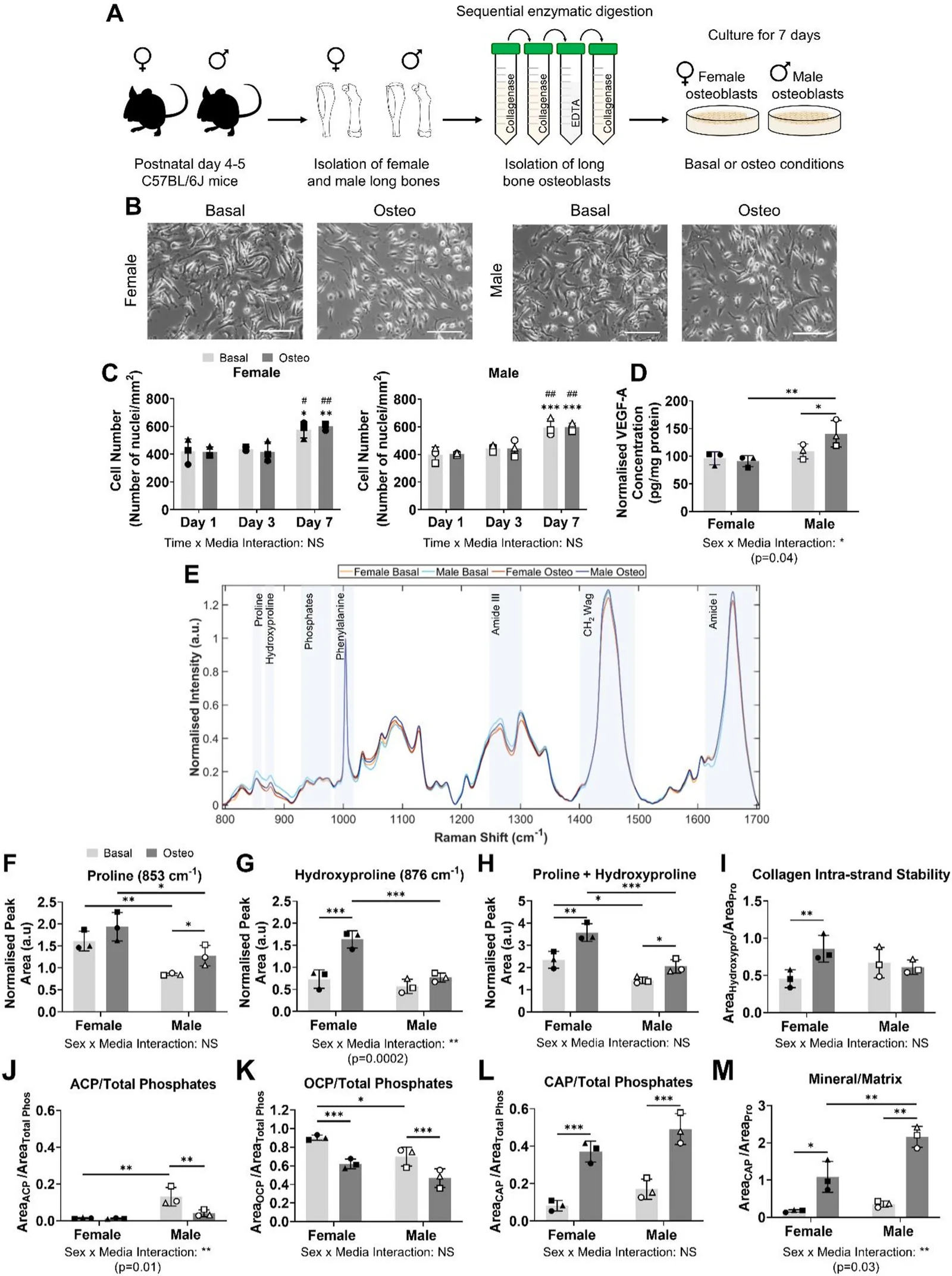
Divergent osteoblast matrix composition and VEGF-A release occur independently of cellular growth profiles. Primary long bone osteoblasts were isolated from 4-5-day old female and male C57BL/6J mice using the collagenase-collagenase-EDTA-collagenase (CCEC) method and cultured for 7 days under basal or osteogenic (osteo) conditions (**A**). Representative phase contrast images show comparable osteoblast morphology across sex and culture conditions at day 7 on quartz coverslips (B; scale bars = 100 μm). Cell number increased over time, with no significant differences between basal and osteo conditions in female or male cultures on day 1, 3 or 7 (**C**). Osteogenic culture increased VEGF-A release in male osteoblast cultures, with levels exceeding those detected in female osteoblast cultures under osteo conditions (**D**). Spectrum-wide differences in normalised Raman class mean spectra generated from 125 spectra per group were observed between female and male osteoblast cultures in bands corresponding to type I collagen-associated components within the ECM, including proline (853 cm^− 1^), hydroxyproline (876 cm^− 1^), the amide III region (1200 cm^− 1^ to 1300 cm^− 1^), CH_2_ wag (1450 cm^− 1^) and amide I (1625 cm^− 1^ to 1680 cm^− 1^), as well as mineral-associated vibrations within the phosphate region from 940 cm^− 1^ to 980 cm^− 1^ (**E**). Spectral deconvolution of class mean spectra highlighted sex- and medium-dependent differences in type I collagen-associated components, including proline (**F**), hydroxyproline (**G**) and their sum (**H**), as well as collagen intra-strand stability (**I**). Further divergence was evident in the mineral precursors of hydroxyapatite, including amorphous calcium phosphate (ACP; J), octacalcium phosphate (OCP; K) and carbonated apatite (CAP; L), which were reflected in the mineral/matrix ratio (**M**). Data are presented as mean ± SD, with symbols representing individual biological replicates. Interactions between sex and media were assessed using repeated-measures two-way ANOVA with Šídák’s post hoc test (*p < 0.05, **p < 0.01, ***p < 0.001, ****p < 0.0001). In panel C, statistical annotations denote differences in cell number relative to day 1 (*p < 0.05, **p < 0.01, ***p < 0.001, ****p < 0.0001) and day 3 (^#^*p* < 0.05, ^##^*p* < 0.01) within each sex

Primary osteoblasts were isolated from the long bones of 4–5 day-old female and male C57BL/6J mouse pups and cultured for 7 days under basal or osteogenic conditions prior to the characterisation of sex-specific ECM composition by Raman spectroscopy (Fig. 1A). As demonstrated previously, Raman spectroscopy enables the detection of matrix and mineral components of the ECM synthesised by primary osteoblasts undergoing differentiation, corresponding with elevated alkaline phosphatase (ALP) activity and positive Alizarin Red staining [41].

At day 7 of culture, no overt sex differences in osteoblast morphology were observed under either culture condition (Fig. 1B), with osteoblasts derived from both sexes consistently adopting a flattened appearance with elongated processes. Growth curve analysis confirmed similar proliferation profiles between female and male osteoblast cultures across timepoints and media conditions, with cell number increasing in both populations over time as expected (Fig. 1C). In both sexes, cell number was greater on day 7 relative to day 1 (female basal, *p* = 0.02; female osteo, *p* = 0.005; male basal, *p* = 0.0004 and male osteo, *p* = 0.0005) and day 3 (female basal, *p* = 0.03; female osteo, *p* = 0.005; male basal, *p* = 0.004 and male osteo, *p* = 0.004). Despite comparable growth, VEGF-A release into CM was comparable between female and male osteoblast cultures under basal conditions (Fig. 1D). Under osteogenic conditions, however, male osteoblasts released 1.55-fold more VEGF-A than female osteoblasts (*p* = 0.01). Assessment of media effects revealed that osteogenic induction did not alter VEGF-A release in female osteoblasts but induced a 1.29-fold increase in VEGF-A release in male osteoblast cultures relative to basal conditions (*p* = 0.05). Two-way ANOVA detected no significant interaction between time and media condition for cell number in male or female osteoblast cultures (Fig. 1C). By contrast, a significant interaction between sex and media condition was detected for VEGF-A release (Fig. 1D).

### Raman spectroscopy reveals sex-specific matrix and mineral signatures of female and male osteoblasts

Raman spectroscopy was performed on osteoblast cultures to characterise ECM composition at day 7. Spectra were acquired from osteoblasts cultured under basal and osteogenic conditions across the “fingerprint region”, spanning 800 cm^− 1^ to 1700 cm^− 1^ (Fig. 1E). Inspection of class mean spectra revealed clear sex differences in Raman peaks and bands corresponding to type I collagen-associated components including proline (853 cm^− 1^) and hydroxyproline (876 cm^− 1^), CH_2_ deformation (1450 cm^− 1^) and amide I (1660 cm^− 1^), as well as mineral-associated phosphate vibrations between 940 cm^− 1^ and 980 cm^− 1^.

Univariate spectral deconvolution of normalised class mean spectra revealed greater proline peak area in female than male osteoblast cultures under both basal (*p* = 0.007) and osteogenic (*p* = 0.02) conditions (Fig. 1F). Osteogenic culture further increased proline peak area only in male cultures (*p* = 0.05). By contrast, hydroxyproline peak area was comparable between the sexes under basal conditions (Fig. 1G) but was greater in female than male osteogenic cultures (*p* = 0.006). Osteogenic induction selectively increased hydroxyproline peak area in female osteoblast cultures (*p* = 0.0004), with no corresponding effect detected in male cultures. The combined proline and hydroxyproline peak area (853 cm^− 1^ + 876 cm^− 1^, Fig. 1H), used as a surrogate for type I collagen present within the ECM, was consistently higher in female than male osteoblasts under both basal (*p* = 0.02) and osteogenic (*p* = 0.0008) conditions. Osteogenic medium also induced elevations in combined peak area in both sexes (female; *p* = 0.005 and male; *p* = 0.05). The hydroxyproline/proline area ratio, used as an indicator of collagen intra-strand stability, did not differ between the sexes under either culture condition (Fig. 1I) and was increased only in female cultures in response to osteogenic stimulation (*p* = 0.004).

To determine whether these sex differences and media-dependent effects extended to Raman bands corresponding to hydroxyapatite mineral precursors, further spectral analysis was performed on the total phosphate region. As a function of the total phosphate region, amorphous calcium phosphate (ACP, 948 cm^− 1^), the earliest detectable precursor of hydroxyapatite, was more abundant in male than female basal osteoblast cultures (*p* = 0.001; Fig. 1J). ACP peak area remained unchanged following osteogenic induction in female cultures and was lower in osteogenic male than basal osteoblast cultures (*p* = 0.005). Conversely octacalcium phosphate (OCP, 970 cm^− 1^), an intermediate and transient hydroxyapatite precursor, was enriched in female compared with male basal cultures (*p* = 0.002; Fig. 1K) and was reduced following osteogenic induction in both sexes (female; *p* = 0.0002 and male; *p* = 0.0005). The peak area of carbonated apatite (CAP, 959 cm^− 1^), representing the final mineral precursor of hydroxyapatite, did not differ between female and male osteoblast cultures under either condition (Fig. 1L), and increased in both sexes in response to culture with osteogenic supplements (female; *p* = 0.0003 and male; *p* = 0.0002). Consistent with these observations, the mineral/matrix ratio (CAP/proline peak area) was comparable between basal female and male cultures, whereas osteogenic male osteoblast cultures had a greater mineral/matrix ratio than female cultures under the same conditions (*p* = 0.002; Fig. 1M). Osteogenic supplementation increased the mineral/matrix ratio in both female (*p* = 0.02) and male (*p* = 0.001) cultures. Two-way ANOVA confirmed significant interaction effects of osteoblast sex and culture media on hydroxyproline peak area, ACP peak area and the mineral/matrix ratio, whereas no interaction effects were detected on proline peak area, the combined proline and hydroxyproline peak area, collagen intra-strand stability ratio, OCP peak area or CAP peak area. Collectively, these spectral analyses reveal inherent differences in osteoblast maturation, with those derived from female bone displaying a collagen-dominant, OCP-enriched matrix signature, whereas male osteoblasts exhibit a comparatively mineral-mature ECM profile, characterised by CAP deposition leading to a higher mineral/matrix ratio.

**Fig. 2:**
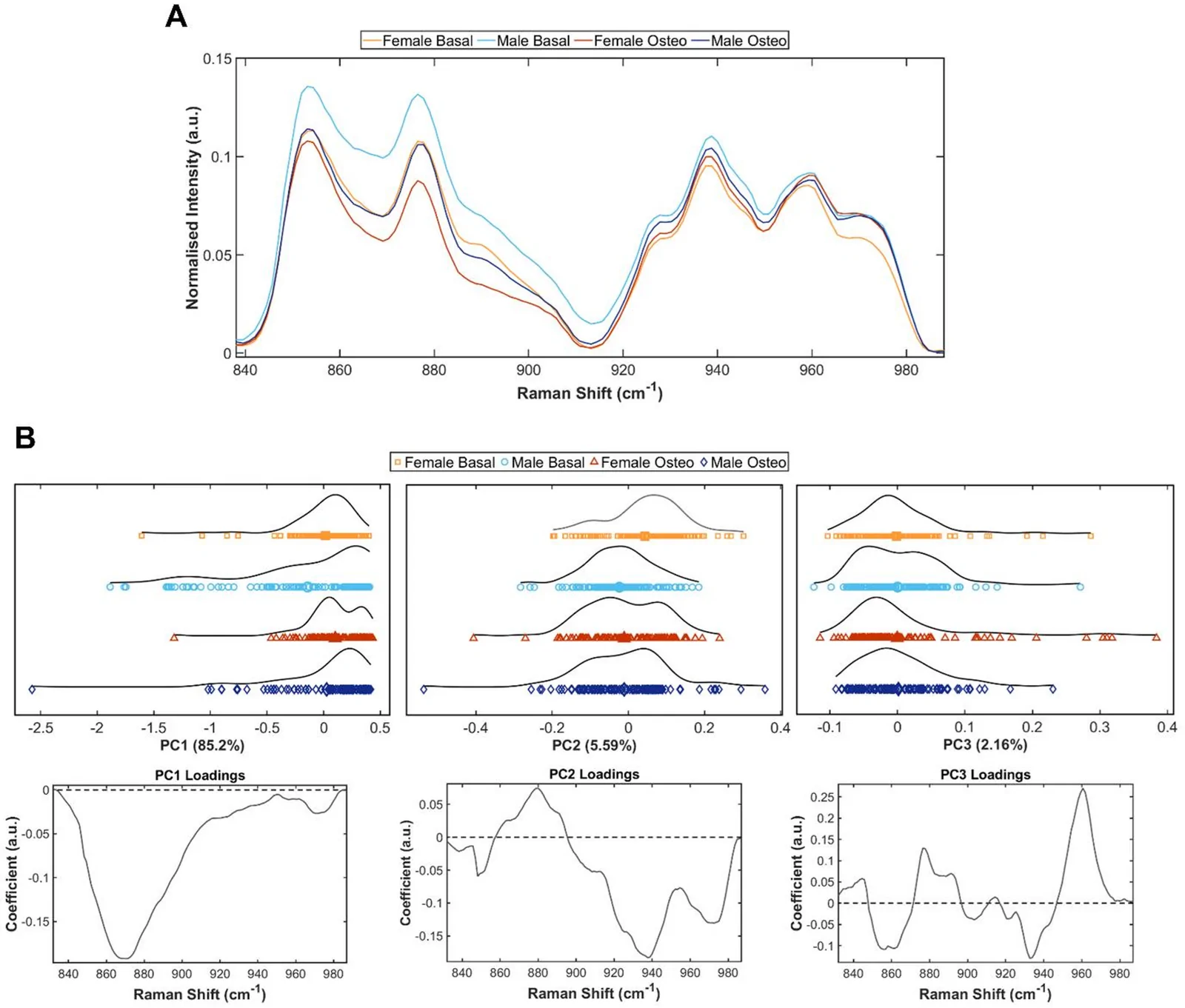
PCA of Raman spectra distinguishes sex- and medium-dependent variation in osteoblast ECM composition. Zoomed class mean Raman spectra show the 840 cm^− 1^ to 980 cm^− 1^ region corresponding to type I collagen-associated and mineral-associated phosphate species (**A**; *N* = 125 spectra per condition). PCA of this region is presented as 1-dimensional score scatter plots showing the distribution of female basal (orange square), female osteogenic (osteo; red triangle), male basal (blue circle) and male osteo (navy diamond) Raman spectra (**B**). Mean PC score is shown as a single larger symbol for each condition, with smaller symbols representing the PC score for individual spectra. Corresponding PCA loading plots, presented underneath, show the loading coefficients for each Raman shift, indicating the spectral regions contributing the greatest variation within each PC

To further interrogate sex-dependent and medium-induced spectral variation, principal component analysis (PCA) was applied to the 840 to 980 cm^− 1^ regions of the class mean spectra (Fig. 2A). PC1 accounted for 85.2% of the total variance and separated spectra according to sex and media culture condition, with separation driven predominantly by the proline-associated peak at ~ 870 cm^− 1^ (C-C stretching mode; Fig. 2B, left). Male basal osteoblast spectra (mean score = -0.14163) clustered distinctly from female basal (PC mean score = 0.0175935), female osteogenic (PC mean score = 0.101326) and male osteogenic (PC mean score = 0.0253243) osteoblast spectra, consistent with biochemical divergence in ECM composition. PC2 accounted for 5.59% of the variance (Fig. 2B, middle) and was primarily influenced by the ~ 879 cm^− 1^ peak of hydroxyproline, distinguishing female basal (PC mean score = 0.0433638) from male basal (PC mean score = -0.0233738), female osteogenic (PC mean score = -0.0107576) and male osteogenic (PC mean score = -0.0105736) osteoblast spectra. Minor contributions from peaks at ~ 848 cm^− 1^, ~ 937 cm^− 1^ and ~ 971 cm^− 1^ corresponding to polysaccharide, protein and C-C wagging modes, respectively, were also observed. PC3 accounted for 2.16% of the variance (Fig. 2B, right) and provided only minor additional separation. Female osteoblast spectra had similar PC scores (basal; PC mean score = -0.0014436 and osteogenic; PC mean score = -0.000250587) but were separated from male basal (PC mean score = 0.000317781) and osteogenic (PC mean score = 0.00159337) spectra. This separation was associated with peaks at ~ 856 cm^− 1^ and 932 cm^− 1^, corresponding to proline and C-C bond vibrations, respectively, in addition to peaks at ~ 877 cm^− 1^, ~ 892 cm^− 1^ and ~ 960 cm^− 1^ corresponding to lipids, phosphodiester and CAP, respectively.

### Contact-mediated survival of BMECs is divergently influenced by osteoblast sex in coculture

**Fig. 3:**
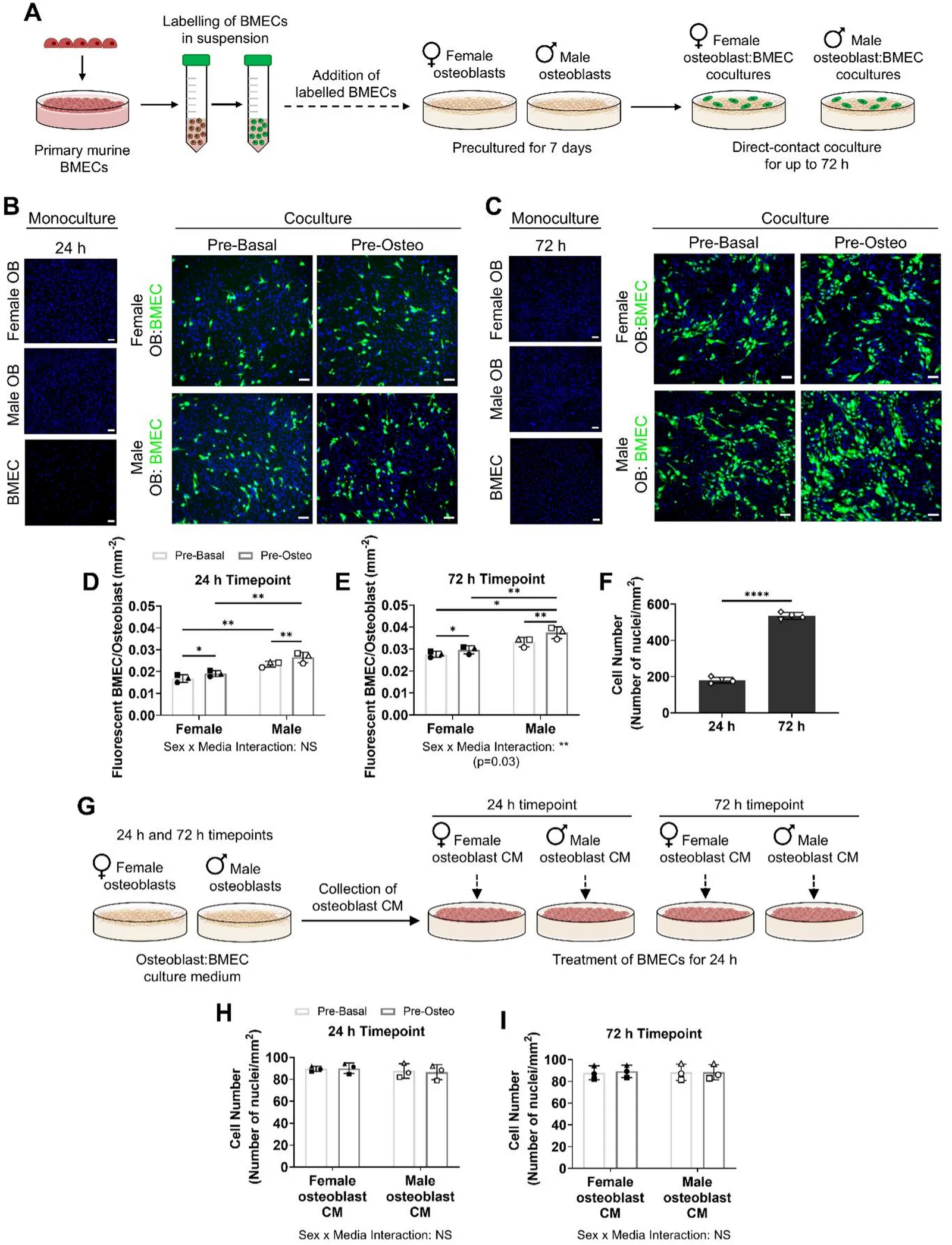
Male osteoblasts support BMEC survival during direct-contact coculture. Primary mouse BMECs were labelled in suspension using CellTracker™ Green prior to heterotypic, direct-contact coculture with female or male osteoblasts at a 1:1 ratio in 50:50 osteoblast: BMEC culture medium for up to 72 h, followed by cell number quantification (**A**). Representative fluorescence images show Hoechst 33258-stained nuclei (blue) in female osteoblast, male osteoblast and BMEC monolayers on day 8 and day 10, corresponding to the 24 h (**B**) and 72 h coculture timepoints (**C**), respectively, alongside corresponding cocultures containing CellTracker™ Green-labelled BMECs (green; scale bars represent 50 μm). Fluorescent BMEC number per osteoblast was increased in cocultures with male osteoblasts after 24 h (**D**) and 72 h (**E**). BMEC monocultures maintained in 50:50 osteoblast: BMEC medium increased in number over time (**F**). For CM experiments, female and male osteoblast monolayers were cultured in basal or osteogenic medium until day 7, after which media were replaced with 50:50 osteoblast: BMEC culture medium for up to 72 h. CM were collected after 24 h and 72 h, corresponding to days 8 and 10, respectively, and used to treat BMEC monocultures for 24 h prior to cell number quantification (**G**). Osteoblast-derived CM from the 24 h (**H**) and 72 h (**I**) timepoints had no effect on BMEC number. Data are presented as mean ± SD, with symbols representing individual biological replicates. Interactions between sex and media were assessed using repeated-measures two-way ANOVA with Šídák’s post hoc test (**D**, **E**, **H** and **I**). Statistical significance between timepoints (**F**) was assessed using Student’s paired t-test (*p < 0.05, **p < 0.01, and ****p < 0.0001)

To determine whether the osteoblast-derived ECM influences endothelial cell behaviour in a sex-specific manner, fluorescently labelled primary murine BMECs were seeded onto established female and male osteoblast monolayers on day 7 and maintained in direct-contact coculture in 50:50 osteoblast: BMEC medium for up to 72 h (Fig. 3A; timeline in Figure S3A) with BMEC expansion assessed by fluorescence microscopy.

Following 24 h of direct-contact heterotypic coculture, sex differences in BMEC number were evident (Fig. 3B), and became more pronounced by 72 h (Fig. 3C). Quantification of cell numbers after 24 h of coculture revealed 1.39-fold greater BMEC numbers in cocultures with male than female osteoblasts, irrespective of whether the osteoblasts were preconditioned with basal medium (*p* = 0.004) or osteogenic medium for 7 days (*p* = 0.002; Fig. 3D). By 72 h, BMEC number remained 1.2-fold greater in cocultures with male osteoblasts preconditioned in basal medium (*p* = 0.02) and 1.26-fold greater in those preconditioned in osteogenic medium (*p* = 0.003) compared with cocultures containing female osteoblasts (Fig. 3E). Assessment of media effects revealed that osteogenic preconditioning was associated with greater BMEC numbers in cocultures with both female and male osteoblasts at 24 h (1.23-fold in female; *p* = 0.02 and 1.13-fold in male; *p* = 0.008) and 72 h (1.07-fold in female; *p* = 0.02 and 1.13-fold in male; *p* = 0.002).

To determine whether these differences were contact-dependent, BMECs were maintained in monoculture for 72 h in 50:50 osteoblast: BMEC medium (Fig. 3F) or treated with CM collected from female and male osteoblast monocultures at equivalent 24 h and 72 h coculture timepoints (Fig. 3G). As expected, BMEC number increased over time in monoculture (*p* < 0.0001; Fig. 3F). However, treatment of BMEC monocultures with CM collected from female or male osteoblasts preconditioned in basal or osteogenic medium did not recapitulate the sex differences in BMEC number observed during direct contact coculture at either timepoint (Fig. 3H and I). In osteoblast monocultures, prior culture in basal or osteogenic medium did not differentially affect cell number between day 1 and day 7, nor did subsequent replacement with 50:50 osteoblast: BMEC media alter osteoblast growth between day 8 and day 10 corresponding to the coculture timepoints (female; Figure S5A and male; Figure S5B). As expected, the number of osteoblasts increased over time in cultures of both sexes between day 1 and day 3 (female basal; *p* = 0.007 and osteo; *p* = 0.01, and male basal; *p* < 0.0001 and osteo; *p* = 0.0003) and day 1 and day 7 (all *p* < 0.0001). Consistent with this, osteoblast numbers at days 7, 8 and 10 were higher than those detected at days 1 and 3 (all *p* < 0.0001). Collectively, these findings indicate that enhanced BMEC number in coculture is contact-dependent, influenced by osteoblast sex, and not reproduced by osteoblast-derived soluble factors alone.

## Discussion

Sexual dimorphism in bone geometry, strength and fracture susceptibility is well established, yet the cellular mechanisms linking osteoblast behaviour to sex-specific organisation of the local vasculature remain incompletely understood. In our previous work, osteoblast-derived VEGF was shown to divergently regulate mineralisation in female and male bone, leading to sexually dimorphic vascular phenotypes at the tibiofibular junction. In the present study, we show that primary murine osteoblasts isolated from female and male bone exhibit distinct ECM signatures that are associated with divergent BMEC responses during heterotypic direct-contact coculture. These findings suggest that intrinsic differences in osteoblast matrix maturation and composition are associated with divergent endothelial cell responses in vitro, providing insight into contact-dependent osteoblast-endothelial interactions that may contribute to sexually dimorphic microenvironments in bone.

**Fig. 4:**
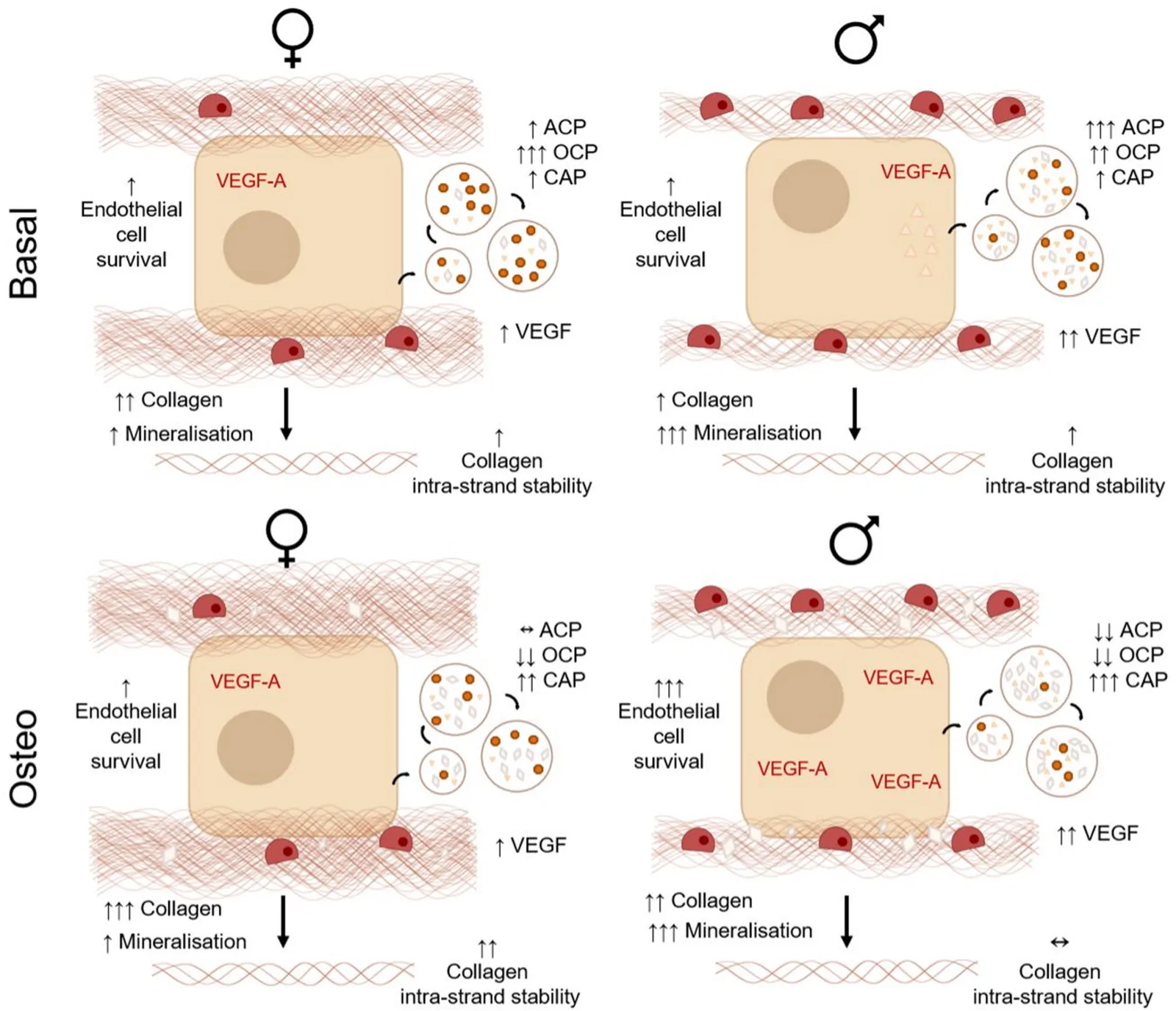
Sex-specific osteoblast matrix signatures are associated with divergent BMEC responses during direct-contact coculture. Raman spectroscopy revealed enrichment of collagen-associated species and OCP in basal female osteoblast cultures (left, top), which shifted towards an early mineralisation-associated profile following osteogenic (osteo) induction, characterised by reduced OCP, increased CAP, increased collagen intra-strand stability and elevated VEGF release (left, bottom). Male osteoblast cultures showed lower levels of collagen-associated species and higher levels of ACP under basal conditions (right, top), progressing towards a more mineral-mature signature under osteogenic conditions, with increased CAP, a higher mineral/matrix ratio and elevated VEGF release (right, bottom). During direct-contact coculture, these osteoblast-derived matrix environments were associated with greater BMEC number in cocultures containing male than female osteoblastss

Using heterotypic coculture models, we demonstrate that osteoblast sex is associated with divergent endothelial cell responses in a contact-dependent manner. Direct-contact cocultures revealed enhanced BMEC survival, reflected by greater BMEC number, in cultures containing osteoblasts derived from male compared with female bone under both basal and osteogenic conditions over 72 h (Fig. 4). Intriguingly, this divergence in BMEC number was not reproduced by treatment with osteoblast-derived CM alone, despite greater VEGF-A release from male osteoblasts under osteogenic stimulation. This indicates that soluble factors alone are insufficient to account for the observed differences in BMEC number and instead implicates osteoblast-derived ECM, local growth factor presentation or direct cell-cell contact as potential contributors to endothelial cell behaviour. This interpretation is supported by evidence that endothelial cell identity and function are actively specified by cell-matrix interactions within the bone microenvironment. In particular, β_1_-integrin mediated adhesion to ECM components such as laminins is required for endothelial survival, differentiation and vascular organisation during developmental osteogenesis, establishing matrix signalling as a key determinant of skeletal angiogenesis [54]. Similarly, the perivascular ECM has been shown to form organ-specific vascular niches that coordinate biochemical and mechanical signalling, integrating growth factor presentation [55] and mechanotransducive inputs to regulate endothelial cell survival [56]. Osteoblast-mediated ECM deposition and mineralisation are therefore not only essential for defining the mechanical competence of bone [57, 58], but also for establishing a bioactive microenvironment that regulates vascular cell fate. In this context, differences in matrix composition, organisation and maturation state may influence endothelial cell behaviour through contact-dependent mechanisms.

To determine whether divergent BMEC responses were associated with differences in osteoblast-derived matrix composition, we employed Raman spectroscopy as this approach enables the simultaneous assessment of organic and inorganic components of the bone matrix [29, 30] and correlates well with bending stiffness compared with conventional measures of bone mineral density (BMD) [59]. Our analyses revealed marked sex differences in compositional ECM signatures, with female osteoblast matrices exhibiting greater type I collagen-associated proline and hydroxyproline alongside enrichment of OCP, consistent with a collagen-associated matrix signature undergoing early mineralisation. In contrast, male osteoblast matrices exhibited lower levels of collagen-associated species with a comparatively more mineral-mature profile, reflected by CAP accumulation and a higher mineral/matrix ratio. Osteogenic induction further revealed divergent maturation kinetics between the sexes: proline increased selectively in male cultures and hydroxyproline in female cultures, while the combined proline and hydroxyproline peak area, CAP and mineral/matrix ratio increased in both sexes and OCP decreased in both sexes. Collectively, these matrix and mineral signatures are consistent with day 7 representing an early matrix maturation window, during which osteoblast-derived matrix deposition and early mineralisation are already evident [38, 60], while enabling spectral profiling of individual cells and reliable assignment of matrix signatures to defined cell populations. Raman spectroscopy is well suited to interrogating this stage, as we and others have shown that it can sensitively detect early proliferative and differentiation-associated changes in primary osteoblast cultures before corresponding changes are readily resolved by conventional ALP activity, osteogenic gene expression or Sirius Red collagen deposition assays [60]. In differentiating osteoblast cultures, Raman spectroscopy has similarly identified spectral profiles associated with matrix formation, including type I collagen species and ACP, and their progression towards mineralisation-associated profiles marked by CAP accumulation, with these shifts corresponding to changes in ALP activity, *Phospho1* expression, Alizarin Red staining, and pro-angiogenic VEGF-A release [41]. The relationship between osteoblast matrix maturation and VEGF release is particularly relevant to osteoblast-endothelial crosstalk, as vascular invasion and endothelial remodelling occur alongside active matrix deposition and mineral maturation [33, 61–63]. Building on these findings, we have previously shown that osteoblast-derived VEGF-A regulates bone vascularisation and matrix mineralisation in a sexually dimorphic manner, and that osteoblast-specific *Vegfa* loss reveals distinct activities of female and male osteoblasts [29, 30]. These sex-specific matrix phenotypes were associated with divergent expression of genes involved in matrix formation and mineralisation, matrix remodelling and angiogenesis, including *Col1a1*,* Spp1*,* Mmp13*,* Sparc*,* Lama1*,* Mmp9* and *Thbs1* [30]. In parallel, osteoblast-derived VEGF-A loss induced sex-specific changes in BMEC expression of angiogenesis-associated genes, including *Igf1*, further implicating divergent osteoblast-endothelial signalling between the sexes [29].

Taken together with our previous molecular evidence of sex-specific osteoblast and angiogenic programming, the present findings suggest that female and male osteoblasts differ intrinsically in their matrix-forming capacities, independent of differences in cell number. Rather than reflecting greater overall matrix abundance, the male osteoblast profile was characterised by a shift towards the maturation of mineral precursor species, whereas female osteoblasts retained a more collagen-associated ECM signature. This interpretation is supported by the comparable hydroxyproline/proline ratios observed between the sexes, indicating that post-translational modifications of collagen, and therefore intra-strand stability, are preserved despite differences in matrix abundance. These compositional differences align with established sex differences in skeletal growth trajectories, whereby males undergo prolonged cortical bone accrual through periosteal expansion while females experience earlier skeletal maturation with preferential endosteal bone formation [1, 64, 65]. In mice, this is further substantiated by reports of reduced expression of *Alpl* and *Bglap*, ALP activity and nodule formation in female osteoblast cultures derived from C57BL/6 mice alongside reduced differentiation potential of bone marrow stromal cells from female C57BL/6 mice compared with males [66]. Critically, the presence of these sexually divergent matrix signatures in vitro, in the absence of circulating sex hormones supports the concept that osteoblasts retain autonomous sex-specific functional programming ex vivo, which has similarly been reported in other bone cell populations including osteoclasts [21, 22] and osteocytes [23, 24].

However, beyond their compositional implications, these sex differences in osteoblast matrix signatures may contribute to divergent vascular responses within the skeletal niche. Despite exhibiting stronger collagen-associated Raman signals, female osteoblast cultures exerted a comparatively weaker effect on BMEC survival than male osteoblast cultures. This observation suggests that collagen abundance alone is insufficient to support BMEC responses observed here and instead implicates additional matrix-associated factors, including mineral-phase composition and VEGF-ECM interactions. Consistent with this, male osteoblast cultures exhibited greater VEGF-A release under osteogenic culture conditions. Given that specific VEGF isoforms bind to ECM components [67, 68], and that growth factor delivery in conjunction with CAP enhances angiogenic responses during bone regeneration [69, 70], it is plausible that the CAP within the matrices synthesised by male osteoblasts may facilitate the local retention or presentation of pro-angiogenic signals at the osteoblast-BMEC interface. Indeed, skeletal angiogenesis is closely coupled to osteogenesis, reflected by the spatial intimacy maintained between osteoblasts and vascular endothelial cells throughout embryonic development and postnatal growth [61, 71]. Osteoblast-derived VEGF functions through paracrine, autocrine and intracrine signalling mechanisms to stimulate endothelial proliferation and vessel formation via VEGF receptor activation [28, 71, 72]. However, endothelial cells also require adhesion to the ECM to support migration, survival and angiogenic organisation. In bone and other tissues, type I collagen has been widely reported to promote angiogenesis both in vitro and in vivo [73–78]. For example, endothelial cells cultured on type I collagen matrices undergo rapid cytoskeletal reorganisation and form branching capillary-like networks [74, 79]. Our findings contrast with this paradigm and instead suggest that endothelial behaviour reflects the integrated biochemical environment of the matrix, including the presentation of pro-angiogenic cues from local cell types and their matrix components.

Although intrinsic sex differences in osteoblast matrix composition have not previously been described, sexually dimorphic regulation of the ECM has been reported across a number of biological systems. For example, sex differences in ECM composition and tissue mechanics have been observed in the brain, where neuroinflammatory ECM remodelling has been shown to contribute to altered cortical viscoelasticity in multiple sclerosis [80, 81]. Similarly, sex-specific matrix remodelling has been reported in cardiovascular tissues, where the ageing male cardiac ECM exhibits enrichment of distinct collagen isoforms associated with pro-fibrotic, injury-like remodelling phenotypes [82, 83]. At the cellular level, sexually divergent ECM regulation has been described in vascular cell populations, including valvular interstitial cells and smooth muscle progenitor cells, where differences in collagen synthesis, matrix remodelling enzymes and proliferative behaviour contribute to sex differences in ECM composition and disease susceptibility [84, 85]. A clearer understanding of the mechanisms governing sex-specific matrix assembly at the cellular level in skeletal tissues is therefore important for elucidating how bone matrix organisation and angiogenic processes are differentially regulated. Investigating these interactions in vivo remains challenging, as the bone matrix is highly mineralised and, consequently, the mechanisms linking osteoblast-derived matrix properties to vascular behaviour have been difficult to study. The matrix signatures described here may provide insight into the mechanisms contributing to sex differences in a number of skeletal conditions including osteopenia [86], osteoarthritis [87], osteoporosis [88] and fracture repair [89], which are increasingly recognised to involve systemic vascular alterations [90, 91]. Defining how osteoblast-vascular crosstalk is modulated by matrix composition may therefore inform more accurate diagnosis and the development of sex-informed therapeutics, biomaterials and regenerative approaches tailored to distinct skeletal microenvironments.

A key limitation of the present study is that the donor sex of the commercially sourced primary murine BMECs was not specified by the supplier. It therefore remains unclear whether BMEC sex influenced the endothelial cell responses observed here, or whether female- and male-derived BMECs would respond differently to osteoblast ECM environments. Future studies should therefore examine sex-matched and sex-mismatched osteoblast-BMEC cocultures using BMECs isolated from female and male mice. Additional limitations of our study include the extensive use of murine cells, the absence of single nucleotide polymorphism genotyping to confirm sub-strain identity while monitoring potential genetic drift in the C57BL/6J colony, and the focus on early matrix formation at day 7 rather than later-stage cultures comprising mineralised nodules. Future studies using human skeletal cells, larger biological replicate numbers and later matrix maturation timepoints will be important to determine whether these sex-specific matrix signatures are conserved across species, confirm their reproducibility, and establish whether they persist during more advanced stages of osteoblast mineralisation.

The molecular mechanisms underlying the sex differences in BMEC number observed during heterotypic, direct-contact cocultures similarly remain to be elucidated. Potential mechanisms may involve differential integrin-mediated adhesion, VEGF receptor signalling, matrix-associated growth factor presentation or matrix stiffness-dependent mechanotransduction linked to the distinct ECM signatures identified here. Future mechanistic studies combining spectroscopic matrix profiling with targeted manipulation of osteoblast-derived ECM composition in sex-matched and sex-mismatched coculture models, alongside transcriptomic analysis of osteoblast and BMEC populations sorted from these cocultures, would enable these candidate pathways to be directly examined and linked to reciprocal changes in angiogenesis, matrix formation and remodelling. Subsequent in vitro studies could then determine how 17β-oestradiol, testosterone or dihydrotestosterone, with or without oestrogen or androgen receptor inhibition, modify osteoblast matrix composition through spectroscopic profiling, VEGF-A release, and BMEC behaviour assays. This would define the relative contributions of intrinsic cellular sex differences and hormone-dependent signalling prior to assessing their interactions during skeletal development and ageing in vivo.

## Conclusions

In summary, this study demonstrates that osteoblasts from female and male mice deposit intrinsically distinct ECMs that are associated with divergent endothelial cell responses through contact-dependent mechanisms. Raman spectroscopic analyses revealed that female osteoblasts generate a type I collagen- and OCP-enriched ECM, whereas male osteoblasts produce matrices with comparatively lower levels of type I collagen-associated components, exhibit CAP accumulation and a higher mineral/matrix ratio indicative of a more mineral-mature ECM profile. These compositional differences were accompanied by enhanced BMEC number in cocultures with male osteoblasts and were linked to greater osteoblast-derived VEGF-A release in this sex, suggesting that sex-specific matrix assembly may contribute to divergent angiogenic microenvironments in bone. By situating these findings within the broader context of sexual dimorphism in skeletal growth and vascular biology, our work identifies osteoblast matrix programming as a cell-autonomous contributor to sex-specific skeletal vascular regulation. Together, these findings provide a means to link nanoscale matrix composition to sexually dimorphic skeletal architectures and may inform the development of sex-informed regenerative therapeutic strategies.

### Perspectives and significance

This study positions osteoblast-derived ECM composition as an important contributor to sex-specific osteoblast-endothelial interactions, highlighting matrix maturation state as a potential mediator of vascular cell behaviour. By integrating heterotypic coculture models with Raman spectroscopy, we demonstrate that compositional differences in the osteoblast-derived ECM are associated with contact-dependent endothelial cell responses in a sex-specific manner. These findings establish Raman spectroscopy as a powerful tool for resolving biologically relevant differences in osteoblast-derived matrix composition and for interrogating how intrinsic cellular sex differences are encoded within the extracellular environment and translated into distinct cell-cell interactions. Future studies combining sex-matched and sex-mismatched coculture models, exogenous hormone and hormone receptor inhibition studies and targeted manipulation of matrix-associated signalling pathways will be essential to determine how these mechanisms contribute to divergent skeletal physiology and pathology, and whether these can be leveraged to develop more precise, sex-informed regenerative strategies.

## Supplementary Information

Below is the link to the electronic supplementary material.

Supplementary Material 1

## Data Availability

The datasets used and/or analysed during the current study are available from the corresponding author on reasonable request.

## Abbreviations

ACP: Amorphous calcium phosphate
ALP: Alkaline phosphatase
ANOVA: Analysis of variance
BMEC: Bone marrow-derived endothelial cell
BMD: Bone mineral density
CAP: Carbonated apatite
CCEC: Collagenase-collagenase-EDTA-collagenase
CM: Conditioned media
DAPI: 4’,6-diamidino-2-phenylindole
DMSO: Dimethylsulfoxide
ECM: Extracellular matrix
EDTA: Ethylenediaminetetraacetic acid
ELISA: Enzyme linked immunosorbent assay
ERα: Oestrogen receptorα
FBS: Fetal bovine serum
FITC: Fluorescein isothiocyanate
HBSS: Hank’s balanced salt solution
OCP: Octacalcium phosphate
PBS: Phosphate buffered saline
PCA: Principal component analysis
SD: Standard deviation
SAGER: Sex and gender equity in research
VEGF-A: Vascular endothelial growth factor A
αMEM: α-Minimum essential medium
