# Supplementary material for: Sex differences in murine bone matrix maturation modulate osteoblast-endothelial interactions
Source: Biol Sex Differ. 2026 Aug 2;17:173. doi: 10.1186/s13293-026-00960-3 (PMC13721271; doi:10.1186/s13293-026-00960-3)
Supplement: Supplementary file 1 [file 13293_2026_960_MOESM1_ESM.docx]

**Supplementary Information**

**Figure S1: Raman spectral processing workflow.** The quartz background spectrum (A), acquired from blank quartz coverslips using identical instrument settings, was manually subtracted from raw osteoblast spectra (B) to generate quartz background subtracted spectra (C) in WiRE 4.1. The resultant spectra, presented as class mean spectra, were subsequently Haar-wavelet denoised in iRootLab (D), baseline corrected using a 9^th^-order polynomial (E), then normalised to the 1004 cm^-1^ peak of phenylalanine (F). Data is presented as class means of N=25 spectra with SD displayed (B-E).

**Figure S2:** Raman spectra acquired across the fingerprint region from 800 cm^-1^ to 1700 cm^-1^ are presented as class mean spectra generated from 125 single spectra per group across three biological replicates, with SD shown, for female basal (A), female osteogenic (osteo; B), male basal (C) and male osteo (D) osteoblasts. Second derivative spectra were generated for each sex and culture condition (E-H) to identify bands of interest, including type I collagen-associated proline (853 cm^-1^) and hydroxyproline (876 cm^-1^), and mineral-associated phosphate species within the 940 cm^-1^ to 980 cm^-1^ region. These band positions were used to guide spectral deconvolution. Representative peak fitting of a class mean spectrum is shown (I), with peaks of interest indicated by opaque curves.

**Figure S3:** Experimental timeline for direct-contact heterotypic coculture studies (A). Female and male primary long bone osteoblasts, isolated from 4-5-day-old C57BL/6J mice, were cultured under basal (red dashed line) or osteogenic (green dashed line) conditions for 7 days. On day 7, culture media were removed and unlabelled or CellTracker™ Green-labelled BMECs were introduced at a 1:1 seeding ratio. Heterotypic cell layers were maintained in direct contact in 50:50 osteoblast:BMEC medium for 24 h to 72 h prior to quantitative analysis. Representative fluorescence images show CellTracker™ Green-labelled BMECs and Hoechst 33258-stained nuclei after 24 h and 72 h of culture (B, left and right, respectively), illustrating decay in CellTracker™ Green fluorescence over time (scale bars=50 µm).

**Figure S4:** Fluorescence microscopy images of Hoechst 33258-labelled nuclei (A) and CellTracker™ Green-labelled BMECs (B) in heterotypic direct-contact cocultures were acquired sequentially from the same field of view, pseudocoloured and overlaid (C; nuclei, blue and BMEC, green). CellTracker™ Green-positive cells were quantified in FIJI by manual counting of green-fluorescent cells containing a visible Hoechst-positive nucleus (D; scale bars=50 µm).

**Figure S5:** Cellular growth profiles of female (A) and male (B) osteoblasts cultured under basal or osteogenic (osteo) conditions until day 7, followed by maintenance in 50:50 osteoblast:BMEC medium until day 10. Cell number increased over time in both female and male osteoblast cultures, with no differences between basal- and osteo-precultured cells on day 7, 8 or 10 in either sex. Data are presented as mean ± SD, with symbols representing individual biological replicates. Interactions between time and media were assessed using repeated-measures two-way ANOVA with Šídák’s post hoc test. Statistical comparisons indicate differences relative to day 1 (*p<0.05, **p<0.01, ***p<0.001, ****p<0.0001) and day 3 (^####^p<0.0001) in each sex.
